# PEA-15 C-Terminal Tail Allosterically Modulates Death-Effector Domain Conformation and Facilitates Protein–Protein Interactions

**DOI:** 10.3390/ijms20133335

**Published:** 2019-07-07

**Authors:** Sergio L. Crespo-Flores, Andres Cabezas, Sherouk Hassan, Yufeng Wei

**Affiliations:** Department of Chemistry, New Jersey City University, Jersey City, NJ 07305-1596, USA

**Keywords:** protein–protein interaction, mitogen-activated protein (MAP) kinase, apoptosis, protein structure, nuclear magnetic resonance (NMR)

## Abstract

Phosphoprotein enriched in astrocytes, 15 kDa (PEA-15) exerts its regulatory roles on several critical cellular pathways through protein–protein interactions depending on its phosphorylation states. It can either inhibit the extracellular signal-regulated kinase (ERK) activities when it is dephosphorylated or block the assembly of death-inducing signaling complex (DISC) and the subsequent activation of apoptotic initiator, caspase-8, when it is phosphorylated. Due to the important roles of PEA-15 in regulating these pathways that lead to opposite cellular outcomes (cell proliferation vs. cell death), we proposed a phosphostasis (phosphorylation homeostasis) model, in which the phosphorylation states of the protein are vigorously controlled and regulated to maintain a delicate balance. The phosphostasis gives rise to the protective cellular functions of PEA-15 to preserve optimum cellular conditions. In this article, using advanced multidimensional nuclear magnetic resonance (NMR) techniques combined with a novel chemical shift (CS)-Rosetta algorithm for de novo protein structural determination, we report a novel conformation of PEA-15 death-effector domain (DED) upon interacting with ERK2. This new conformation is modulated by the irregularly structured C-terminal tail when it first recognizes and binds to ERK2 at the d-peptide recruitment site (DRS) in an allosteric manner, and is facilitated by the rearrangement of the surface electrostatic and hydrogen-bonding interactions on the DED. In this ERK2-bound conformation, three of the six helices (α2, α3, and α4) comprising the DED reorient substantially in comparison to the free-form structure, exposing key residues on the other three helices that directly interact with ERK2 at the DEF-docking site (docking site for ERK, FxF) and the activation loop. Additionally, we provide evidence that the phosphorylation of the C-terminal tail leads to a distinct conformation of DED, allowing efficient interactions with Fas-associated death domain (FADD) protein at the DISC. Our results substantiate the allosteric regulatory roles of the C-terminal tail in modulating DED conformation and facilitating protein–protein interactions of PEA-15.

## 1. Introduction

In recent years, the multifaceted interactions and numerous biological pathways that are involved and regulated by phosphoprotein enriched in astrocytes-15 kDa (PEA-15), a death effector domain (DED) containing protein, have drawn significant attention in the fields of cancer, diabetes, and neurodegenerative diseases [1,2]. PEA-15 prevents membrane association of insulin-sensitive glucose transporter 4 (GLUT4) through the interaction with phospholipase D1 and D2 (PLD1/2) [3], which activates protein kinase C (PKC)-α and –β, and blocks insulin-induction PKC-ζ activity [4]. PEA-15 overexpression is considered a common defect in first-degree relatives of type 2 diabetic patients, and is associated with reduced insulin sensitivity in these individuals [5]. PEA-15 inhibits Fas/TNF (tumor necrosis factor)-α induced apoptosis by binding to the adapter protein FADD (Fas-associated death domain) in the death-inducing signaling complex (DISC) and blocking the recruitment and activation of procaspase-8 [6,7,8]. PEA-15 deactivates extracellular signal-regulated kinase 1 and 2 (ERK1/2) in the mitogen-activated protein (MAP) kinase pathway in a Ras-dependent manner [9]. When PEA-15 binds to ERK, it promotes cytosolic localization of ERK and blocks ERK-dependent transcription and proliferation [10,11]. It has been demonstrated that PEA-15 can protect vascular smooth muscle cells from inappropriate proliferation through the interaction and cytosolic localization of ERK1/2 [12]. However, phosphorylation at both Ser^104^ and Ser^116^ of PEA-15 abrogates its ability to prevent nuclear translocation of ERK1/2 in vivo and in vitro [13]. It appears that phosphorylation at Ser^104^ blocks ERK binding, and Ser^116^ phosphorylation promotes recruitment of PEA-15 into the DISC, inhibiting apoptosis [14]. Phosphorylation of PEA-15 seems to switch PEA-15 from a tumor-suppressor to a tumor-promoter [15]. The phosphorylation of PEA-15 and the induced dissociation from ERK2 have also been implied in the regulation of the suprachiasmatic nucleus (SCN) timing system of the hypothalamus [16].

PEA-15 forms a tight 1:1 complex with ERK2 [17] with a dissociation constant, *K*_d_, in sub-micromolar (μM) range independent of phosphorylation states of either PEA-15 or ERK2 [18]. Structurally, PEA-15 possesses a canonical six-helix bundled DED and an irregularly structured C-terminal tail [19,20]. Recent nuclear magnetic resonance (NMR) studies suggested that PEA-15 utilizes its DED residues from helices α1, α5, and α6 to bind to ERK2 [21], while the other three helices α2, α3, and α4 undergo substantial conformational change, but are not directly involved in binding [22]. Recent crystal structures of PEA-15/ERK2 complex confirmed most findings of our NMR studies, including the binding interface between PEA-15 and ERK2, and identified a few key residues on helix α5 that are directly involved in binding [23]. However, the crystal structures could not fully resolve residues at helices α2, α3, and α4 of PEA-15 as this region becomes very flexible and disordered upon ERK2 binding [21]. In this study, we employed de novo structural generation protocol, Rosetta, coupled with experimentally determined backbone NMR chemical shifts (CS), as implemented in the Biological Magnetic Resonance Data Bank (BMRB) CS-Rosetta Server [24,25,26,27], to characterize the unique structural features of the PEA-15 DED in its complex with ERK2. In the complex, PEA-15 DED adopts a novel conformation distinctive from the free-state structure. In particular, the helices α2, α3, and α4 are completely rearranged and reoriented from the free-form positions, and helix α3 becomes very flexible and randomly structured, whereas the other three helices, α1, α5, and α6, overlap fairly well between the free and ERK2-bound structures.

Additionally, we have explored the allosteric effects of C-terminal phosphorylation at S104 and S116 on the overall protein conformation by introducing serine to aspartic acid mutations at both residues. A previous study suggested that the S104D/S116D double mutant (PEA-15DD) has similar in vivo effects as the doubly phosphorylated form in inhibiting apoptosis and promoting cell adhesion, migration, and proliferation by selectively interacting with FADD [28]. Our initial NMR results illustrate that the PEA-15DD adopts a conformation divergent from either the wild-type protein or the ERK2-bound form, while its complex with FADD shows moderate shifts in NMR spectrum to that of the PEA-15DD only, suggesting that the C-terminal tail acts as an allosteric modulator in determining the DED conformations and its binding specificity.

## 2. Results

### 2.1. CS-Rosetta Models of PEA-15 in Free and ERK2-Bound Forms

**Figure 1:**
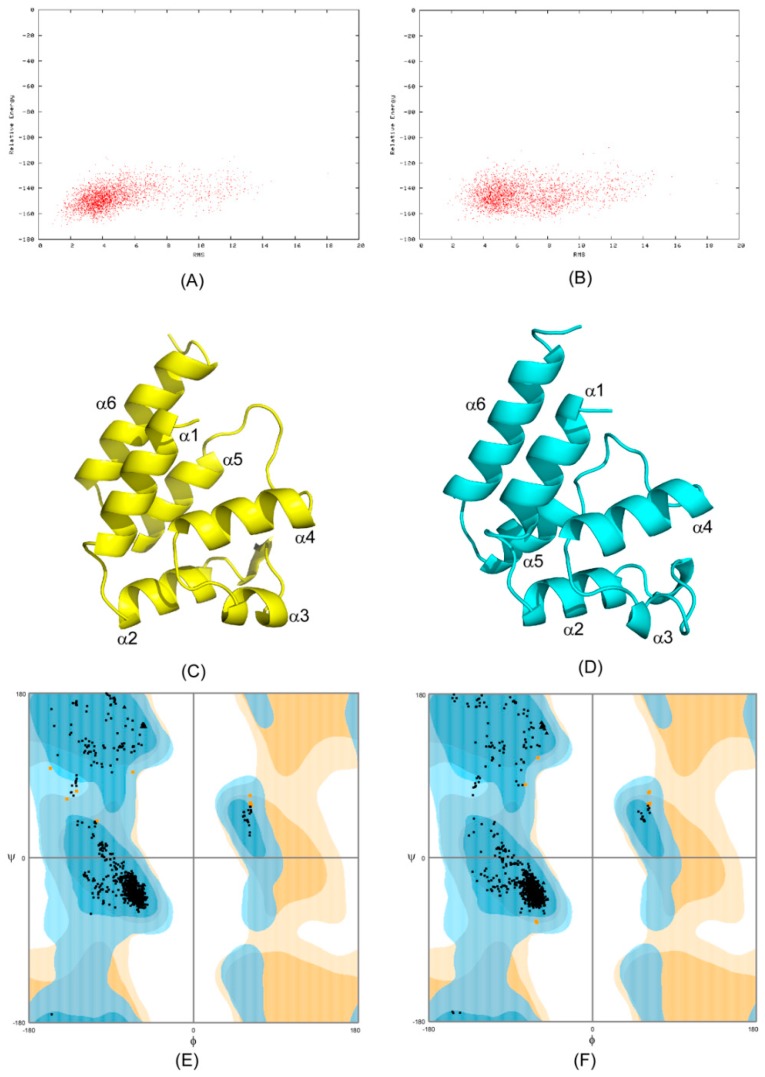
Chemical shift (CS)-Rosetta de novo structural generation for phosphoprotein enriched in astrocytes-15 kDa (PEA-15) in free and extracellular signal-regulated kinase 2 (ERK2)-bound form. (**A**) Plot of Rosetta energy versus Cα root-mean-square deviation (RMSD) for the free form. (**B**) Plot of Rosetta energy versus Cα RMSD for the ERK2-bound form. (**C**) Lowest energy structure of PEA-15 in free form. (**D**) Lowest energy structure of PEA-15 in ERK2-bound form. (**E**) Ramachandran plot for free-form PEA-15. (**F**) Ramachandran plot for the ERK2-bound PEA-15. Both Ramachandran plots show 98.9% of residues in a favored region (black dots), 1.1% in an allowed region (orange dots), and no residues in a disallowed region.

Using CS-Rosetta de novo structural calculation protocol [24,25,26,27], we generated 3000 DED (residues 1–90) structures for each of the free and ERK2-bound forms of PEA-15 using backbone HN, N, Cα, and Cβ chemical shifts. The plots of Rosetta all atom energy versus Cα root-mean-square deviation (RMSD) indicated convergence of the calculations for both forms (Figure 1A,B). The ten best structures with the lowest overall energies were selected for both free and ERK2-bound PEA-15, with the lowest-energy ones shown in Figure 1C,D. Both free and ERK2-bound forms of PEA-15 consist of a six-helix bundled structure of a typical death-effector domain, but the relative orientations of certain helices are quite distinctive between the two structures. In addition, the helix α3 becomes more random and flexible upon ERK2 binding. The geometries of both free and ERK2-bound forms were assessed using Ramachandran plots calculated from RamPage, a web-based geometrical validation program [29]. In both structural ensembles, 98.9% of all residues are located in a favored region, 1.1% in an allowed region, and no residues in a disallowed region (Figure 1E,F).

**Figure 2:**
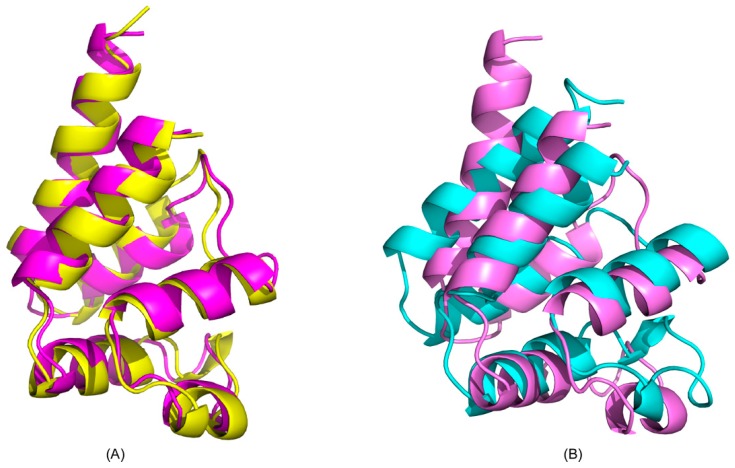
CS-Rosetta structural models superimposed with published structure of PEA-15 (Protein Data Bank (PDB) ID 2SL7). (**A**) Superimposition of the free-form structure (yellow) with 2LS7 (magenta). (**B**) Superimposition of the ERK2-bound form structure (cyan) with 2LS7 (magenta).

To validate and confirm the structures generated from CS-Rosetta, we compared the free-form CS-Rosetta model of PEA-15 with our previously published high-definition NMR structure calculated using a full set of NMR restraints, including chemical shifts, nuclear Overhauser effects (NOEs), dihedral angles, and residual dipolar couplings, and further refined with an explicit solvent protocol [20]. The CS-Rosetta structural ensembles closely resemble the published structure (Protein Data Bank (PDB) ID: 2LS7), with the average backbone RMSD between the 10 best structures and 2LS7 being 2.42 ± 0.77 Å. Figure 2A shows a representative CS-Rosetta structure overlaid with 2LS7 (RMSD = 1.406 Å). In this overlay, all six helices in the DED are well aligned with published structure, demonstrating the feasibility of using sparse NMR restraints, such as backbone chemical shifts in this case, coupled with Rosetta protocol to generate de novo structures for PEA-15. Due to the flexibility of the C-terminal tail of PEA-15, residues 91–130 were not included in the calculations and structural generations, although the full-length protein was used in NMR data collection. In the ERK2-bound form, however, the DED structure is significantly different from the published free-form structure (Figure 2B), with the average backbone RMSD between the 10 best structures and 2LS7 being 6.29 ± 3.01 Å. The discrepancies between the CS-Rosetta generated free and ERK2-bound structures are similar, with average backbone RMSD 6.09 ± 3.06 Å.

### 2.2. Recognition of ERK2 by PEA-15 C-Terminal Tail Allosterically Induces Conformational Change at the DED

**Figure 3:**
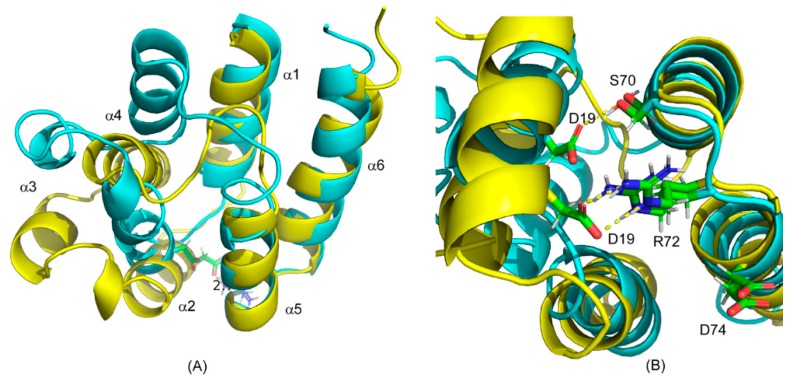
Superposition of CS-Rosetta structural models of the free and ERK2-bound forms of PEA-15. (**A**) Superimposition of the free form (yellow) with the bound-form models (cyan). The ERK2-bound PEA-15 displays minimum changes in helices α1, α5, and α6, but significant conformational change in helices α2, α3, and α4. (**B**) Positions of charge-triad residues in the free (yellow) and ERK2-bound models (cyan). The D19 residue moves significantly, while the R72 and D74 residues mostly stay at their original positions. The hydrogen-bonding interactions between the D19 and R72 residues in the free-form structure is replaced by the D19–S70 hydrogen bond in the ERK2-bound structure.

A more detailed analysis of the free and ERK2-bound structures reveals that the structural discrepancies between the two forms result from the reorientation of helices α2, α3, and α4, while the other three helices α1, α5, and α6 largely retain their conformations and orientations (backbone RMSD 1.501 Å), although helix α5 is shortened by about half turn (Figure 3A). The short unwinding of helix α5 is due to the fact that a few key residues on this helix are directly involved in ERK2 binding, including E68, I69, and R71 [23], and possible involvement of S70 in reorganizing surface polar interactions and facilitating conformational change (see Section 3 below). Compared with the free-form structure, ERK2-bound PEA-15 displays the most significant conformational change in helices α2, α3, and α4. Helix α2 rotates about 16° from its free-form orientation, and helices α3 and α4 move about 6.6 and 5.3 Å, respectively, from their free-form positions. In addition, helix α3 becomes a lot shorter and more randomly structured. These observations are consistent with a previous NMR dynamic study, which has revealed that, while the C-terminal tail becomes more rigid and less flexible at fast timescale upon binding to ERK2, many DED residues display increased flexibility and more complicated motions at both fast and slow timescales [21]. At the very end of the PEA-15 C-terminal tail, residues 121–129 contain a d-peptide sequence in a reversed order [18,21,30], which recognizes and binds to the ERK2 D-peptide recruitment site (DRS, also called DEJL domain). Spectral density analysis indicated that the fast motion in the d-sequence region has been greatly reduced upon ERK2 binding, while a significant number of DED residues exhibit both increased fast motions and elevated chemical exchange (*R*_ex_) at a slower timescale [21]. Our CS-Rosetta PEA-15 model for the ERK2-bound form confirms the findings of the NMR dynamics study. Helices α2, α3, and α4 become more randomly structured and flexible, corroborating the increased fast motion in this region, and the switching to new orientations and conformation in the bound form leads to enhanced *R*_ex_. The strong correlation of the dynamic behaviors in the DED and the C-terminal tail upon ERK2 binding has largely established the allosteric nature of controlling DED conformational change by the C-terminal tail. In fact, deletion of the D-peptide sequence from PEA-15 completely sabotages its ability to bind to ERK2 [19], substantiating the cooperative roles of the C-terminal tail.

### 2.3. Charge-Triad Residues Putatively Modulate Conformational Change of the DED

Nearly all death-effector domains possess a characteristic surface feature, the charge triad, D/E-RxDL (x representing any amino acid), in an extended electrostatic and hydrogen-bonding network [31]. In PEA-15, the charge triad is composed of D19, R72, and D74 residues that have been shown to be hydrogen-bonded together in its free from [20]. Two of the charge-triad residues, R72 and D74, located on the loop between helices α5 and α6 and on helix α6, respectively, do not move in any significant way between the free-form and ERK2-bound PEA-15, while the D19 residue, located on helix α2, moves about 5.9 Å away from its free-form position upon ERK2 binding. The movement of D19 could prompt the rotation of helix α2 away from its original free-form orientation, and subsequently provoke the conformational changes in helices α3 and α4. The movement of the D19 residue also causes the break of the hydrogen bond between D19 and R72 as seen in the free-form conformation, and the lowest-energy CS-Rosetta model indicates that it forms a new hydrogen bond with S70 on the helix α5 in the ERK2-bound form (Figure 3B). Previous crystal structures of PEA-15/ERK2 complexes also showed that D19 is no longer in close contact with R72. When full-length PEA-15 was co-crystalized with the T185E ERK2 mutant, D19 was shown to be hydrogen-bonded to K259 on ERK2, while the truncated PEA-15 (residues 1–96) complexes with unphosphorylated or doubly phosphorylated ERK2 did not partake in D19 interactions [23]. The inconsistency is apparently due to the fact that this region of PEA-15 is of high dynamic complexity, with increased fast motions and elevated chemical exchanges among various conformers and is involved in multiple modes of the interactions with ERK2. D19 has been demonstrated to be an important residue in ERK2 interaction. The mutation of D19 to arginine (D19R) disrupts the interaction between PEA-15 and ERK2 [23]. D19 exhibits fairly large chemical shift perturbation (CSP) between the free-form protein and the ERK2 complex, and it possesses increased motions and flexibility at a fast timescale [21]. Interestingly, residue S70 is dominated by slow-motion *R*_ex_ in its dynamic profile, making it a good candidate to get involved in the new interactions with D19, albeit the nature of the interactions involving D19 and S70 entails further scrutiny.

### 2.4. PEA-15 C-Terminal Tail Phosphorylation Allosterically Modulates DED Conformation to Accommodate FADD Binding

**Figure 4:**
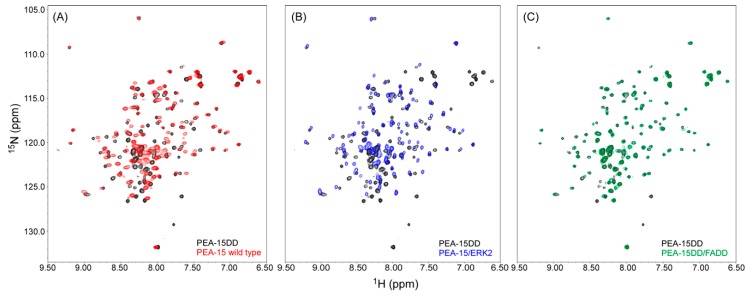
Two-dimensional (2D) heteronuclear single-quantum coherence (HSQC) nuclear magnetic resonance (NMR) spectrum of the PEA-15 S104D/S116D double mutant (PEA-15DD) (black) superimposed with the spectra of (**A**) the wild-type PEA-15 (red); (**B**) the wild-type PEA-15 in the complex with ERK2 (blue), and (**C**) PEA-15DD in the complex with Fas-associated death domain (FADD) (green).

Two-dimensional (2D) heteronuclear single-quantum coherence (HSQC) NMR spectroscopy allows quick assessment of protein conformational changes and protein–protein or protein–ligand interactions by observing the resonance (peak) shifts, as we previously reported the profound conformational change of PEA-15 upon ERK2 binding [21]. The PEA-15DD double mutant, mimicking the doubly phosphorylated form of the protein, displays an NMR spectrum with many resonances shifted from the spectra of the wild-type and ERK2-bound form of PEA-15 (Figure 4A,B, respectively). In fact, the spectral change involves about half of all peaks, preventing us from simply relaying the resonance assignments from the wild-type protein to the double mutant. On the other hand, the PEA-15DD spectrum is similar to that of the PEA-15DD/FADD complex, with only moderate shifts in a few resonances caused by the interactions between the two proteins (Figure 4C). This observation indicates that phosphorylation (or aspartate mutation) at the S104 and S116 residues is sufficient to alter the conformation of the protein, making it most suited to bind to FADD.

The C-terminal tail of PEA-15, although flexible and unstructured in nature, plays critical roles in allosteric regulation of PEA-15 protein–protein interactions. It contains an essential signature sequence for the recognition of ERK2 DRS at the very end of the protein (residues 121–129), as well as two phosphorylation sites at S104 and S116. Our earlier NMR dynamics data demonstrated that recognition of ERK2 DRS by the C-terminal signature sequence induces a conformational change in the DED, allowing optimal interactions between PEA-15 and ERK2 [21]. Here, we have provided additional evidence that phosphorylation at the two serine residues on the C-terminal tail can lead to another distinct conformation that gives rise to the FADD binding specificity. Combining these experimental evidences together, we have concluded that the PEA-15 C-terminal tail allosterically modulates protein structure and binding specificity. The coherent conformation changes among various structures are facilitated by rearranging surface polar and charged residues, including the charge-triad, D19–R72–D74, in forming and breaking hydrogen-bonding and electrostatic interactions among the side chains. As shown earlier, D19 shifted to a new position in the PEA-15/ERK2 complex, breaking the hydrogen bond with R72, and forming a new interaction with another polar amino acid, S70.

## 3. Discussion

Although the significant structural variations between the free and ERK2-bound forms are expected based on our previous NMR studies, it is the first time we have the structural insight into the biologically relevant conformation of PEA-15 in the ERK2 complex. Using protein dynamics [21] and residual dipolar coupling (RDC) data [22], we established an allosteric model that a substantial conformational change in PEA-15 DED is induced by the C-terminal tail and mediated by the charge-triad residues upon ERK2 binding. In this study, we have, for the first time, presented the actual structural models that delineate the details of the orientational and conformational changes in the PEA-15 DED upon ERK2 binding, as well as the rearrangement of surface polar interactions in mediating the conformational change. These structural characteristics were not reported in the previous crystal structures as the relevant regions became too flexible to be observed in crystallography [23]. In our previous high-definition PEA-15 structure, we detected a significant number of surface polar and charged residues forming an extensive network of hydrogen-bonding and electrostatic interactions [20]. We hypothesized that these polar interactions are critical in PEA-15 conformational modulations in that the changes in conformations are energetically compensated for and kinetically facilitated by reorganizing these interactions [22]. The previous crystallographic study of PEA-15 interactions with ERK2 reported that D19 of PEA-15 is no longer involved in the charge-triad interactions [23]. In this study, we have provided the first indication that D19 on helix α2 interacts putatively with S70 on helix α5, which is in agreement with the NMR dynamic properties of these residues. However, the increased fast motions and chemical exchanges involved in these residues could complicate the interactions and could even be transient in nature. The new interaction involving D19 could drive the reorientations of helices α2, α3, and α4, and at the same time destabilize α3 to be more randomly structured.

The conformational changes of PEA-15 DED, mediated by surface polar and charged interactions, are evidently regulated allosterically by the structurally irregular C-terminal tail. Our previous NMR dynamics study already demonstrated that the d-peptide sequence located at the end of the PEA-15 C-terminal tail first recognizes and binds to the DRS on the ERK2 protein, and this interaction induces conformational change at the DED [21]. In this study, we have detailed the changes of the α2, α3, and α4 helices. As reported in the earlier crystal structures of PEA-15/ERK2 complexes, the binding pocket for PEA-15 DED is located on the ERK2 DEF-docking site (docking site for ERK, FxF), and directly interacts with the ERK2 activation loop comprising the phosphorylation sites, E185 and Y187 [23]. The rotation and destabilization of helices α2, α3, and α4 illustrated in this study can greatly facilitate the docking of PEA-15 helix α5 to the ERK2 DEF site by exposing key residues, including E68, I69, and R71 on PEA-15, available for ERK2 binding.

The phosphorylation sites on the PEA-15 C-terminal tail at the S104 and S116 residues are additional allosteric regulators for the DED structure. Our initial NMR assessment has found that the phosphorylation at the two residues, mimicked by S to D mutations, is sufficient to alter the protein conformation. This new conformation is distinctive from both free-form and ERK2-bound structures. The C-terminal tail phosphorylation prepares the protein for accepting FADD as its preferred binding partner through DED–DED homotypic interactions. This is consistent with a cell culture study where S104 phosphorylation blocks ERK2 binding, and S116 phosphorylation promotes its binding to FADD and recruitment into the DISC [14]. Another important interaction of PEA-15 with phospholipase D (PLD) appears to be dependent on S116 phosphorylation [32], and we speculate that this interaction is also regulated allosterically through the C-terminal tail to modulate the overall protein conformation.

## 4. Materials and Methods

### 4.1. Protein Expression and Purification

Full length PEA-15 and FADD proteins were expressed and purified as described previously with some modifications [21]. Briefly, each protein was separately cloned into the Novagen pET-28b vector (EMD Millipore, Burlington, MA, USA) between the restriction sites NdeI and BamHI, and was expressed as hexahistidine (His_6_) tagged protein at the N-terminus. Uniformly ^2^H/^15^N and ^2^H/^13^C/^15^N labeled protein was overexpressed in *Escherichia coli* BL21(DE3) cells in ^2^H/^15^N and ^2^H/^13^C/^15^N labeled *E.coli*-OD2 rich growth media (Silantes GmbH, Munich, Germany), while unlabeled proteins were overexpressed in Lysogeny broth (LB) media. Cells were grown at 37 °C to an optical density O.D. 600~0.4–0.7 and then induced with 1 mM isopropyl-β-d-thiogalactopyranoside (IPTG) for 4 hours. The cells were suspended in bacterial cell lysis buffer (GoldBio, St. Louis, MO, USA) in 1:10 ratio (1 g wet cell pellet in 10 mL of buffer) and lysed according to the manufacture’s protocol. The protein was then isolated from the cell lysate by Co^2+^ affinity chromatography with a HiTrap TALON crude column and subsequently purified to homogeneity by anion-exchange chromatography using a Mono Q 5/50 GL column on an Akta Pure 20L fast protein liquid chromatography (FPLC) system (GE Healthcare Life Sciences, Marlborough, MA, USA).

### 4.2. Mutagenesis

The PEA-15 S104D/S116D double mutant (PEA-15DD) protein was produced by direct point mutations using QuikChange Lightning Site-Directed Mutagenesis Kit (Agilent, Santa Clara, CA, USA) and following the manufacture’s protocol. The mutagenic primers were designed using the web-based QuikChange Primer Design program provided by the manufacture. The mutagenic polymerase chain reactions (PCR) were performed using the manufacture’s recommended program. The mutated plasmid sequence was confirmed by a DNA sequencing service provided by Eurofins Genomics (Louisville, KY, USA). The mutated plasmid was transformed back into BL21(DE3) *E. coli* bacterial cells for expression and purification following the same procedures as described in the previous subsection.

### 4.3. NMR Spectroscopy

The free PEA-15 NMR sample was prepared at 0.7 mM protein in 10 mM sodium phosphate buffer pH 7.0, 1 mM dithiothreitol (DTT) and 50 μM NaN_3_ in 90%/10% H_2_O/D_2_O. The PEA-15/ERK2 complex was formed using the uniformly labeled PEA-15 with unlabeled ERK2 that was expressed and purified as described [33]. The complex NMR sample was prepared with 0.7 mM uniformly labeled PEA-15 and a stoichiometric amount of ERK2 (1:1 molar equivalent) in 10 mM sodium phosphate buffer pH 7.0, 150 mM NaCl, 1 mM DTT, and 50 μM NaN_3_ in 90%/10% H_2_O/D_2_O.

The NMR sample for the free PEA-15DD double mutant protein was also prepared at 0.7 mM protein in in 20 mM sodium phosphate buffer pH 7.0, 150 mM NaCl, 1 mM DTT, and 50 μM NaN_3_ in 90%/10% H_2_O/D_2_O. The PEA-15DD/FADD complex was formed using the uniformly labeled PEA-15DD with unlabeled FADD in the same buffer. The molar ratios between PEA-15DD and FADD were varied from 1:0.5, 1:1, and 1:2. The spectra did not change significantly at 1:1 and 1:2 ratios (data not shown), suggesting a 1:1 binding stoichiometry. All subsequent NMR experiments were performed using 1:1 molar ratio between the two proteins.

All NMR experiments were acquired at 25 °C on Bruker Avance 500 and 800 MHz NMR spectrometers, operating at 1 H frequencies of 500.13 and 800.23 MHz, respectively, and equipped with either a triple-resonance cryoprobe or a regular triple-resonance probe at the New York Structural Biology Center (NYSBC) and Columbia University Chemical Instrumentation Facility. A set of triple resonance backbone assignment experiments were performed to assign PEA-15 backbone chemical shifts [34,35]. All NMR spectra were processed with NMRPipe [36] and analyzed using NMRViewJ [37].

### 4.4. De Novo Structure Generation

De novo structural generations with sparse backbone chemical shifts for PEA-15 in its free form and ERK2-bound form were submitted to the CS-Rosetta Server (https://csrosetta.bmrb.wisc.edu/csrosetta/submit) hosted on the Biological Magnetic Resonance Data Bank (BMRB). Both free and ERK2-bound form calculations were converged. In each calculation, 3000 structures were generated, and the 10 best structures with the lowest overall energy were selected. The RMSD among the 10 best structures was 1.846 ± 0.602 Å for the free form and 2.898 ± 0.441 Å for the bound form. The Ramachandran plots were produced using the web-based program, RamPage, to validate protein geometry [29]. The coordinates of both free and ERK2-bound structures of PEA-15 were deposited into the Protein Data Bank (PDB) with IDs 6P6B and 6P6C, respectively, and the chemical shifts used in the CS-Rosetta calculations for both the free and bound form were deposited into the BMRB with IDs 30613 and 30614, respectively.

## 5. Conclusions

In this research, we have established that the C-terminal tail is the allosteric structural regulator and binding facilitator of PEA-15 in recognizing and interacting with various binding partners. The status of the C-terminal tail modulates the DED structure, as illustrated in our CS-Rosetta model of the ERK2-bound form and our initial NMR results for PEA-15DD and its complex with FADD. The C-terminal tail, either bound to ERK2 or phosphorylated, sends a signal to the DED to rearrange its conformation to accommodate the potential binding partners, and ultimately determines the binding specificity of the protein. The reorganization of the helices in DED is modulated by the rearrangement of hydrogen-bonding and electrostatic interactions among the polar and charged residues on the DED surface. This result is significant because it provides structural insight on the importance of phosphorylation homeostasis in affecting cellular processes and cell fate [38].

## Abbreviations

PEA-15	Phosphoprotein enriched in astrocytes, 15 kDa
ERK	Extracellular-signal regulated kinase
FADD	Fas-associated death domain
DED	Death-effector domain
NMR	Nuclear magnetic resonance
DISC	Death-inducing signaling complex
PDB	Protein Data Bank
BMRB	Biological Magnetic Resonance Data Bank
RMSD	Root-mean-square deviation
